# Fabrication of Microphase-Separated Tröger’s Base Polymer Membranes for Oxygen Enrichment

**DOI:** 10.3390/membranes16010009

**Published:** 2025-12-30

**Authors:** Chaoyue Yang, Li Zhou, Qian Zhang, Ya Huang, Peixiao Zhang, Jingwen Xue, Qing Li, Weijie Sun, Jiayou Liao

**Affiliations:** 1 Research Institute of Natural Gas Technology, PetroChina Southwest Oil and Gasfield Company, Chengdu 401147, China; 2 National ReDo Center for High Sulfur Gas Exploitation, Chengdu 610599, China; 3 High Sulfur Gas Exploitation Pilot Test Center, China National Petroleum Corporation, Chengdu 610599, China; 4 College of Chemistry and Chemical Engineering, Yingxi Campus, Taiyuan University of Technology, Taiyuan 030024, China; 5 China Petroleum Planning and Engineering Institute, Beijing 100120, China

**Keywords:** microphase separation, Tröger’s base polymer, quaternization, gas separation, oxygen enrichment

## Abstract

Tröger’s base (TB) polymers have received increasing attention as a novel class of polymers with intrinsic microporosity, particularly for applications in gas separation. In this study, TB was quaternized with hydrophobic long chains to create a microphase-separated structure to enhance gas separation performance. On one hand, the tertiary amine structure of TB enabled facile grafting modification through the Menshutkin reaction. On the other hand, microphase-separated channels were created in the quaternized Tröger’s base (QTB) membrane due to the polarity differences between the hydrophilicity of the quaternary ammonium groups and hydrophobicity of iodoalkanes, providing channels for gas transport within the membrane and thereby improving permeability selectivity. The successful synthesis of QTB membranes was confirmed by FTIR and ^1^H NMR spectroscopy, while AFM and SAXS analyses validated the microphase-separated morphology. To investigate the impact of microphase separation on oxygen permeability and selectivity, different iodoalkanes and various concentrations of iodobutane were grafted onto the TB backbone. Among the prepared membranes, QTB-C_4_-70% membrane exhibited the highest in O_2_ permeability. Gas separation performance under different O_2_ pressures and temperatures revealed that O_2_ permeability decreased slightly with increasing pressure, indicating good pressure stability of the membrane. With increasing temperature, the permeability increased while the selectivity decreased. These findings demonstrated that microphase-separated QTB membranes offer a viable strategy for creating effective materials for gas separation.

## 1. Introduction

Oxygen-enriched combustion is an energy-efficient technology that enhances fuel utilization and increases flame temperature [1]. When combined with carbon dioxide (CO_2_) capture, this approach can significantly reduce carbon emissions by increasing the CO_2_ concentration in exhaust streams, thereby simplifying impurity removal and reducing the complexity of downstream CO_2_ capture processes [2,3]. Consequently, oxygen-enriched air plays an important role in both daily life and chemical industries.

Conventional gas separation technologies mainly include pressure swing adsorption and cryogenic distillation [4,5,6]. Although these methods can produce high-purity gases on an industrial scale, they suffer from high energy consumption and substantial capital costs. In contrast, membrane-based gas separation has attracted increasing attention due to its low energy demand, reduced capital investment, operational simplicity, and environmental friendliness [7,8,9,10,11]. Among various membrane materials, polymeric membranes have been more widely applied than inorganic counterparts because of their low cost, facile processability, favorable mechanical properties, and good chemical stability. However, with the rapid advancement of industrial processes, conventional polymeric membranes have become increasingly inadequate to meet the growing demands of modern separation applications [12,13,14,15,16,17,18].

Polymers of intrinsic microporosity (PIMs) have been recognized as highly promising microporous polymer materials, exhibiting remarkable characteristics such as good thermal stability, excellent solubility in polar aprotic solvents, well-defined pore structures, and tunable post-modification properties, which originate from the molecular-level designability of their synthetic monomers [19,20,21,22]. Tröger’s Base (TB) polymers represent a novel class of intrinsically microporous polymers with bridged bicyclic ring structures. They have exceptional potential as gas separation membranes due to the rigid N-heterocyclic moieties, which maintain a V-shaped spatial configuration and effectively hinder interchain packing, thereby generating a higher intrinsic microporosity [23,24,25,26,27,28,29,30,31,32]. The inefficient packing of these contorted polymer chains provides microporous free volume that facilitates rapid gas transport, while their extreme rigidity imparts selectivity between different gas species [33,34,35,36,37,38,39,40].

Despite these advantages, TB membranes, like other polymer membranes, are still subject to the well-known trade-off between permeability and selectivity, as described by Robeson’s upper bound [33,41,42]. To improve both permeability and selectivity, strategies such as developing new backbone materials and increasing interchain spacing are often required. Moreover, the structure of TB polymers can be tuned by selecting different diamine monomers or incorporating bulky side groups to enhance gas transport properties [43]. For instance, the McKeown group prepared a polymer with high molecular weight and excellent mechanical properties by designing a three-dimensional rigid monomer for the TB reaction [24]. Wang et al. significantly enhanced the overall separation performance of polyimide membranes by introducing TB moieties into the polymer backbone [44].

Recent studies have indicated that microphase-separated structures can further improve gas transport in membranes [45,46]. By carefully designing the interactions between different phases, microphase separation can create channel networks with large specific surface areas, providing faster pathways for gas molecules and thus enhancing diffusion and transport. This microphase separation approach is expected to serve as an effective strategy for developing high-performance gas separation membranes. In this work, we further aimed to enhance membrane permeability through controllable functionalization modifications.

In this study, we proposed the synthesis of TB polymers using aromatic diamine (4,4′-diamino-3,3′-dimethylbiphenyl) as the starting material. Utilizing the tertiary amine structure of TB, which can be readily modified via quaternization reaction, we synthesized functionalized TB derivative membranes with varying carbon chain lengths and contents. By introducing hydrophobic long-chain alkyl groups through halogenated alkyl compounds, the polarity differences between the hydrophilic quaternary ammonium groups on the main chain and the hydrophobic side chains are expected to induce the formation of a microphase-separated structure. This phase separation was expected to create well-defined gas transport channels, thereby enhancing permeability and selectivity. The experimental design involves altering the carbon atom count in haloalkanes and the reaction feed ratios to prepare a series of structurally tunable derivatives, aiming to achieve gas separation membranes with both high selectivity and permeability. Finally, gas permeation tests were conducted to evaluate the gas separation performance of the prepared membranes, providing insights into the effects of microphase separation on oxygen transport properties.

## 2. Experiment

### 2.1. Materials

All chemicals were used as received unless otherwise specified. Iodoethane, 1-iodobutane, 1-iodohexane, 1-iodooctane, 1-iodoheptane, and 4,4′-diamino-3,3′-dimethylbiphenyl (98%) were purchased from Aladdin (Shanghai, China). Aqueous ammonia (28%), dichloromethane (99%), and dimethoxymethane (DMM) were also obtained from Energy Chemical (Anqing, China). N-Methyl-2-pyrrolidone (NMP), methanol, and trifluoroacetic acid (TFA) were acquired from local suppliers (Taiyuan, China) and used without further purification. Distilled water was prepared in-house using a laboratory distillation system.

### 2.2. Synthesis of TB Polymer

**Scheme 1:**
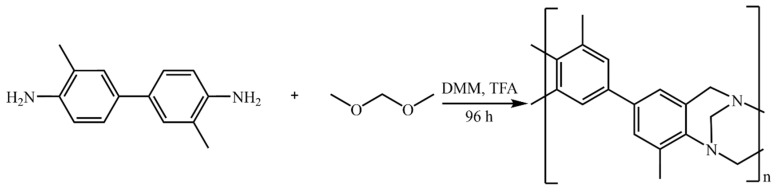
Synthesis of TB.

The TB polymer was prepared according to previous reports [24,32], and the detailed procedure is illustrated in Scheme 1. A mixture of diethoxymethane (35.84 g, 471.1 mmol) and 4,4′-diamino-3,3′-dimethylbiphenyl (20 g, 94.21 mmol) was placed in a 1000 mL three-neck round-bottom flask equipped with a mechanical stirrer. After 15 min, the reaction mixture was cooled to 0 °C using an ice-water bath. Trifluoroacetic acid (TFA, 160 mL) was then added dropwise while maintaining the temperature at 0 °C. After the complete addition, the reaction was allowed to proceed at room temperature (25 °C) under continuous stirring for 96 h, during which the solution gradually increased in viscosity. The crude product was purified by sequential treatment with aqueous ammonium hydroxide and repeated washing with deionized water. Further purification was achieved through solvent-exchange precipitation: the polymer solution in dichloromethane was slowly poured into vigorously stirred methanol. This precipitation procedure was repeated three times to ensure complete purification. The resulting polymer was collected and air-dried at ambient temperature for 24 h, followed by vacuum drying at 100 °C for an additional 24 h to remove residual solvents. The final product was obtained as a white, fibrous material with a fluffy texture.

### 2.3. Quaternized Modification

**Scheme 2:**
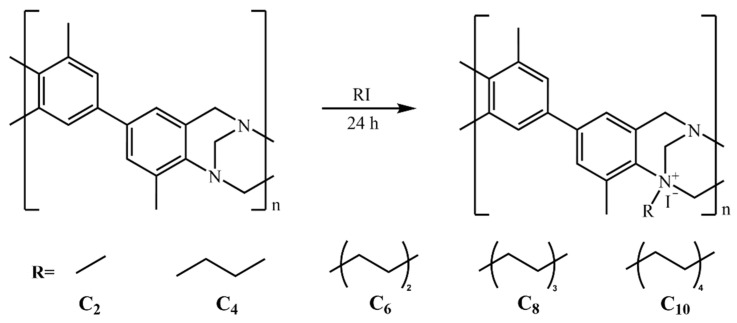
Synthesis of QTB.

The quaternized polymers (QTB) were synthesized following an established Scheme 2 [32]. To investigate the structure–activity relationship between side-chain length and gas transport properties, quaternization reactions were performed using hydrophobic alkyl halides of varying chain lengths. The TB polymer was functionalized with iodoethane (C_2_), 1-iodobutane (C_4_), 1-iodohexane (C_6_), 1-iodooctane (C_8_), and 1-iododecane (C_10_) at the same quaternization concentration. The resulting membranes were labeled QTB-C_2_-70%, QTB-C_4_-70%, QTB-C_6_-70%, QTB-C_8_-70%, and QTB-C_10_-70%, where the suffix indicates the number of carbon atoms in the alkyl chain. To optimize the quaternization concentration, specific molar ratios of TB to hydrophobic alkyl halides (1:0.3, 1:0.5, 1:0.7, and 1:0.9) were employed. The resulting samples were labeled QTB-C_4_-30%, QTB-C_4_-50%, QTB-C_4_-70%, and QTB-C_4_-90%, allowing identification of the optimal quaternization level.

As an example, the quaternized modification with 1-iodobutane was performed as follows: TB polymer membrane (2.0 g) was dissolved in NMP (20 mL), and 1-iodobutane (1 mL) was added to the solution. The mixture was stirred at 25 °C under a dark atmosphere for 24 h. After the reaction, the product was precipitated by pouring the solution into excess methanol, followed by washing. The polymer was air-dried at 25 °C for 24 h and subsequently dried under vacuum for an additional 24 h. Other quaternized polymers were prepared using the same procedure with different hydrophobic alkyl halides. After the reaction, the products were precipitated in excess methanol, thoroughly washed, and dried under ambient and vacuum conditions as described above. Using this established protocol, a series of QTB membranes with various side-chain lengths were successfully obtained, with consistent reaction parameters applied throughout.

### 2.4. Preparation of Membranes

TB and QTB were in NMP with concentrations (10%) at room temperature under magnetic stirring for 24 h. The obtained solution was left to degas and poured on a glass plate dried in an oven for 24 h. The membranes obtained were then dried under vacuum to eliminate all traces of residual solvents.

### 2.5. Characterization

The ^1^H NMR spectra of the TB polymer were recorded on a Bruker AVANCE III 400 MHz spectrometer (Bruker, Karlsruhe, Germany) using CDCl_3_ as the solvent. Fourier transform infrared (FTIR) spectroscopy was performed using a TENSOR spectrometer (Bruker, Karlsruhe, Germany) to identify chemical functional groups in the range of 4000–400 cm^−1^. Thermal stability was investigated using a Perkin-Elmer TGA-4 thermogravimetric analyzer (PerkinElmer, Waltham, MA, USA) under a nitrogen atmosphere at a heating rate of 10 °C/min from 50 °C to 800 °C. The surface and cross-sectional morphologies of the membranes were examined by scanning electron microscopy (SEM). For cross-sectional imaging, the samples were fractured in liquid nitrogen, sputter-coated with gold, and then observed using a JEOL JSM-7610F SEM (JEOL Ltd., Tokyo, Japan). Microphase separation in QTB membranes was characterized by atomic force microscopy (AFM) using a Dimension Icon system (Bruker, Santa Barbara, CA, USA) with a scan area of 1 µm × 1 µm, and by small-angle X-ray scattering (SAXS) using a Pilatus 2M detector (Dectris Ltd., Baden-Daettwil, Switzerland) at a sample-to-beam distance of 1.54 m. SAXS measurements were performed at beamline 7.3.3 (10 keV) of the Advanced Light Source (ALS) and at beamline BL16B1 (12 keV) of the Shanghai Synchrotron Radiation Facility.

The density of the membranes was determined using a helium pycnometer based on the gas displacement principle. Prior to measurement, all membrane samples were thoroughly dried under vacuum at elevated temperature to remove residual solvent and moisture. A known mass (*m*) of each dried membrane sample was accurately weighed and placed into the pycnometer chamber. Helium gas was then introduced into the chamber, and the sample volume (*V_He_*) was calculated from the pressure changes using the ideal gas law. Due to the small atomic size and inert nature of helium, the measured volume corresponded to the skeletal volume of the polymer matrix. The experimental density (*ρ*) of the membranes was calculated according to:

(1) $$\rho \;=\;\frac{m}{V_{He}}$$

where *m* is the mass of the membrane sample and *V_He_* is the skeletal volume determined by helium pycnometry. Each measurement was repeated at least three times, and the average value was reported to ensure reliability.

The fractional free volume (FFV) of the membranes was subsequently estimated from the experimental density using the group contribution method. The skeletal density ($\rho_{0}$) of the polymer was calculated from the van der Waals volume of the repeating unit:

(2) $$\rho_{0}\;=\;\frac{M}{V_{sp}}$$

where *M* is the molecular weight of the repeating unit and *V_sp_* is the specific van der Waals volume calculated from group contribution parameters. The *FFV* was then determined using the following relationship:

(3) $$FFV\;=\;1\;-\;\frac{\rho }{\rho_{0}}$$

### 2.6. Grafting Ratio Calculation by Spectroscopic Method

Since the characteristic peaks of the grafted monomer and the polymer backbone did not overlap, this study directly compared the integral area of the characteristic peak of the grafted monomer with that of the polymer backbone. The grafting ratio was calculated using the following formula:

(4) $$R\left(\%\right)\;=\;\frac{I_{g}}{I_{0}}\;\times \;\frac{M_{0}}{M_{g}}\;\times \;100$$

where *R* was the Grafting Ratio, *I_g_* was the integral area of the characteristic peak of the grafted monomer, *I*_0_ was the integral area of the characteristic peak of the QTB polymer backbone, *M*_0_ was the molecular weight corresponding to the characteristic peak of the QTB polymer backbone, *M_g_* was the molecular weight corresponding to the characteristic peak of the grafted monomer.

### 2.7. Gas Permeation Test

The pure gas permeability (*P*) of the membrane samples was measured using the well-established time-lag method, following procedures reported in the literature. Prior to testing, each membrane was securely mounted in the permeation cell, and the system was thoroughly degassed under vacuum to remove residual gases. Permeability measurements for N_2_ and O_2_ were performed at 25 °C and an upstream pressure of 50 psi using a constant-volume/variable-pressure apparatus. The gas permeability coefficients were calculated from the steady-state region of the pressure–time curve according to the following equation:

(5) $$P\;=\;\frac{273.15\;\times \;10^{10}}{760}\;\times \;\frac{Vl}{AT\;\times \;\frac{76}{14.7}\;\times \;p_{0}}\;\times \;\frac{dp}{dt}$$

Here, *P* is the gas permeability in Barrer (1 Barrer = 1 × 10^−10^ cm^3^(STP)·cm/(cm^2^·s·cmHg)), *V* is the volume of the downstream chamber (cm^3^), *l* is the membrane thickness (cm), *A* is the effective membrane area (cm^2^), *T* is the measurement temperature (K), *P*_0_ is the upstream pressure (psi), and *dP/dt* is the rate of pressure increase in the steady-state region. The ideal selectivity (*α_x/y_*) between pure components *x* and *y* is defined as the ratio of their respective gas permeability coefficients:

(6) αxy = pxpy

## 3. Results and Discussion

### 3.1. Structure of TB and QTB Membranes

**Figure 1:**
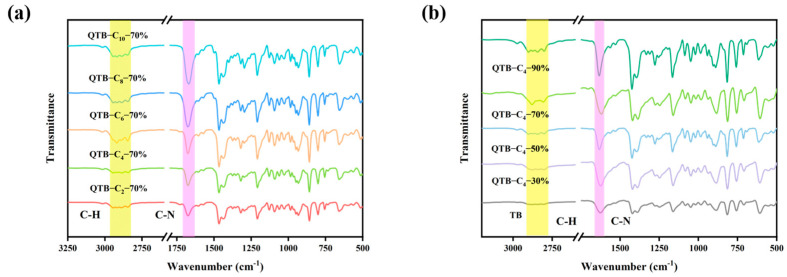
FTIR spectrum of pristine TB and QTB membranes. (**a**) grafted with different lengths of iodoalkanes; (**b**) grafted with different ratios of iodobutane.

Figure 1 shows the FTIR spectra of the pristine TB and QTB membranes prepared with different iodoalkanes and varying concentrations of iodobutane. In the pristine TB membrane, characteristic peaks associated with the bridged bicyclic TB ring include new aliphatic C–H stretching bands in the range of 2947–2842 cm^−1^ [47] and the C–N stretching band at around 1670 cm^−1^ [47]. Furthermore, as the side-chain length increased (Figure 1a) and the iodobutane concentration increased (Figure 1b), the intensities of the C–H (yellow band) and C–N (purple band) stretching vibrations also increased, indicating successful grafting of the side chains.

**Figure 2:**
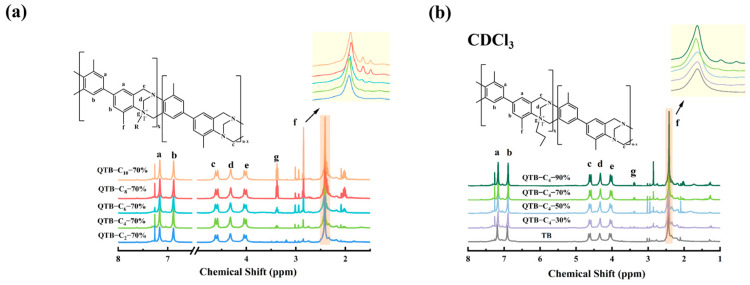
^1^HNMR spectra of pristine TB and QTB in CDCl_3_ membranes. (**a**) grafted with different lengths of iodoalkanes. (**b**) grafted with different ratios of iodobutane. Peak assignments: a and b: protons on the aromatic ring (Ar-H); c, d, e: protons on the saturated heterocyclic ring; g: protons of the α-methylene group in the iodoalkane; f: methyl group linked to the benzene ring.

^1^H NMR spectra of TB and QTB polymers with different iodoalkanes and varying iodobutane concentrations were shown in Figure 2a,b. In the pristine TB polymer, three peaks at 3.9–5.3 ppm, labeled c, d, and e, were attributed to protons of the saturated heterocyclic rings, confirming the successful synthesis of TB. After quaternization with iodoalkanes, a new peak (g) appeared at around 3.38 ppm, corresponding to the methylene protons at the α position of the iodoalkanes, indicating successful quaternization. The degree of quaternization was calculated from the integral area ratio of the characteristic peaks at 3.90–5.30 ppm (c, d, e) and 3.38 ppm (g) for polymers modified with different iodoalkanes. As shown in Figure 2a, the quaternization degrees of QTB-C_2_-70%, QTB-C_6_-70%, QTB-C_8_-70%, and QTB-C_10_-70% were 12.8%, 12.8%, 13.7%, and 12.3%, respectively, confirming the successful modification of TB membranes. Similarly, for membranes prepared with varying iodobutane concentrations (Figure 2b), the quaternization degrees of QTB-C_4_-30%, QTB-C_4_-50%, QTB-C_4_-70%, and QTB-C_4_-90% were 8.7%, 10.2%, 12.6%, and 15.1%, respectively.

### 3.2. Thermogravimetric Analysis

**Figure 3:**
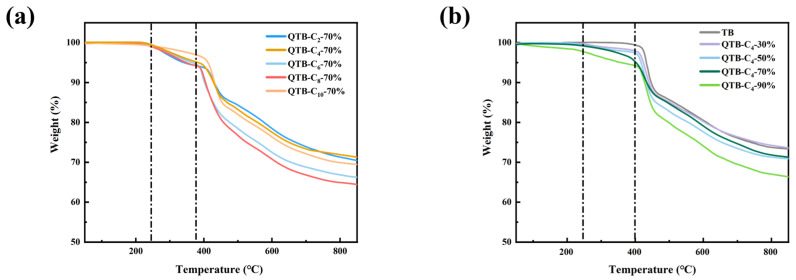
TGA curves of pristine TB and QTB membranes. (**a**) grafted with different lengths of iodoalkanes; (**b**) grafted with different ratios of iodobutane.

The thermal properties of the TB and QTB membranes were investigated by thermogravimetric analysis (TGA). As shown in Figure 3, three distinct weight-loss stages can be identified for the QTB membranes. In the first stage, a minor weight loss observed below approximately 200 °C is mainly associated with the relatively low thermal stability of the introduced quaternary ammonium groups, which may involve cleavage of C–N^+^ bonds and the release of volatile alkyl fragments [48]. The second weight-loss stage occurring in the temperature range of about 250–400 °C can be attributed to the progressive decomposition of alkyl side chains and quaternary ammonium functionalities introduced during the quaternization process. The major and rapid weight loss observed at higher temperatures (above 400 °C) corresponds to the thermal decomposition of the Tröger’s base polymer backbone [49,50].

The weight losses of the QTB-C_2-_70%, QTB-C_6_-70%, QTB-C_8_-70%, and QTB-C_10_-70% membranes were 6.43%, 5.81%, 5.86%, and 4.12%, respectively (Figure 3a). Similarly, the weight losses of the QTB-C_4_-30%, QTB-C_4_-50%, QTB-C_4_-70%, and QTB-C_4_-90% membranes were 2.16%, 2.73%, 3.39%, and 4.15%, respectively (Figure 3b). The weight loss observed after 400 °C was consistent with the trends observed in the aforementioned QTB membranes.

### 3.3. Membrane Morphology

**Figure 4:**
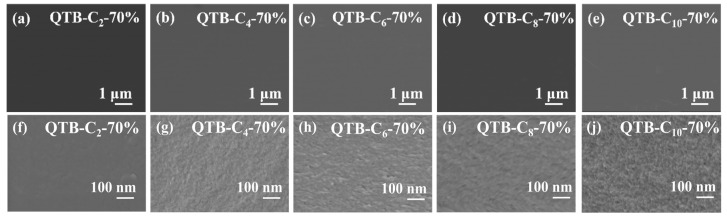
Surface (**a**–**e**) and cross-sectional (**f**–**j**) SEM images of QTB membranes grafted with different lengths of iodoalkanes.

**Figure 5:**
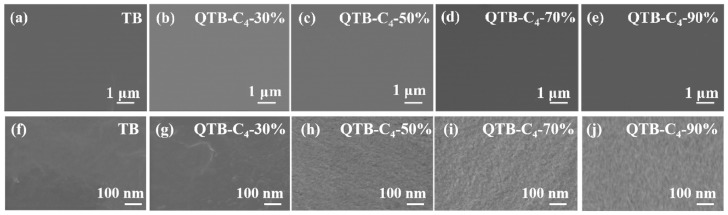
Surface (**a**–**e**) and cross-sectional (**f**–**j**) SEM images of pristine TB and QTB membranes grafted with different ratios of iodobutane.

The surface and cross-sectional morphologies of the TB and QTB membranes were examined by SEM. As shown in Figure 4 and Figure 5, both TB and QTB membranes exhibited dense, homogeneous surfaces without obvious defects. Compared with the pristine TB membranes, quaternization did not induce significant morphological changes, indicating that the QTB membranes maintained excellent film-forming ability. From the cross-sectional images, all membranes displayed compact structures without visible voids or cracks. However, a gradual increase in cross-sectional roughness was observed for the QTB membranes with increasing alkyl chain length and higher alkylation degree. This behavior was attributed to the introduction of flexible alkyl side chains, which disrupted the regular packing and ordering of the polymer backbones. As the alkyl chain length increased and the alkylation degree became higher, this disruption effect became more pronounced, leading to less ordered chain packing and increased morphological heterogeneity within the membrane matrix.

### 3.4. Microphase-Separated Morphology

#### 3.4.1. AFM Analysis

**Figure 6:**
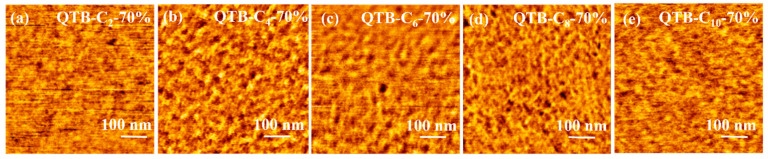
AFM images of QTB membranes grafted with different lengths of iodoalkanes.

**Figure 7:**
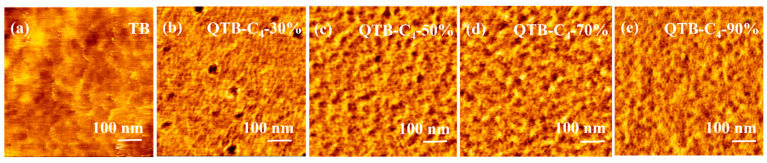
AFM images of pristine TB and QTB membranes grafted with different ratios of iodobutane.

The structure of the TB and QTB membranes was investigated by AFM, as shown in Figure 6 and Figure 7. Compared with the pristine TB membrane, the QTB membranes exhibited a microphase-separated morphology, characterized by distinct dark and light regions. These contrasting domains reflected the formation of mixed phases, confirming the presence of hydrophilic and hydrophobic segments. It had been reported that differences in hydrophilicity and hydrophobicity drove microphase separation, resulting in microscopic pore structures [51], which could enhance gas transport. As shown in Figure 6, the channels in the microphase-separated structure initially widened and then narrowed with increasing length of the grafted carbon chains. The initial widening was attributed to the increased polarity difference between the hydrophilic and hydrophobic segments. However, as the carbon chain length further increased, excessively long flexible aliphatic chains tended to stack and intertwine, leading to the narrowing of channels and gradual blurring of the microphase separation structure.

For the variation in grafting ratio (Figure 7), increasing the butyl iodide grafting ratio from 30% to 70% enhanced the microphase-separated structure, with channels gradually widening. This widening was attributed to the increased polarity difference between the hydrophilic quaternary amine groups and the hydrophobic side chains. When the grafting ratio increased from 70% to 90%, the channels narrowed and the microphase separation became more compact. This effect was caused by excessive side chains interlocking, which led to tighter packing of the polymer chains and blurring of the microphase-separated structure. These results indicated that optimizing both the grafting ratio and the side-chain length was essential for achieving well-defined microphase separation and superior membrane performance.

#### 3.4.2. SAXS Analysis

**Figure 8:**
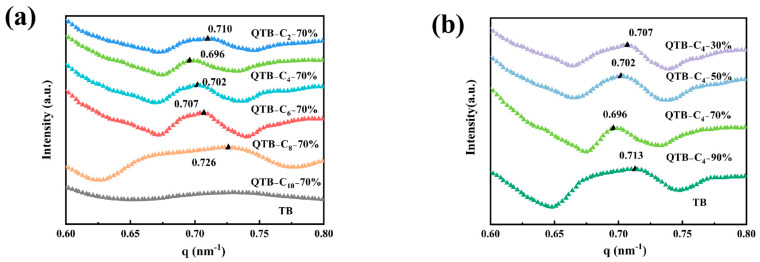
SAXS of pristine TB and QTB membranes (**a**) grafted with different lengths of iodoalkanes; (**b**) grafted with different ratios of iodobutane.

Small-angle X-ray scattering (SAXS) was used to further examine the morphological characteristics of QTB polymers with grafted side chains, in order to elucidate the formation of microphase-separated structures within these polymers. As shown in Figure 8a,b, SAXS analysis revealed distinct scattering peaks for all QTB polymers, whereas the TB polymer exhibited no significant peaks. This observation provided definitive evidence of microphase separation in the QTB polymers.

The inter-domain correlation distance was computed using the Bragg equation (d = 2π/q_max_), with the d-spacing values for QTB polymers, derived from the scattering maxima (q_max_) [52]. These d-spacing values corresponded to the dimensions of the inter-nanodomain distances within the QTB polymers. As depicted in Figure 8a, as the grafted carbon chain length increases, the d-spacing values corresponding to the QTB membranes were 8.845 nm, 9.023 nm, 8.946 nm, 8.883 nm, and 8.650 nm, respectively, showing an initial increase followed by a decrease. This trend was attributed to the enhanced polarity contrast between hydrophilic and hydrophobic segments, which promoted microphase separation and expanded interdomain distances. However, excessive chain length introduced higher chain flexibility, resulting in interchain stacking and entanglement, which reduced microphase separation and decreased d-spacing. Similarly, as the grafting ratio of iodobutane increased from 30% to 70%, the d-spacing increased, reflecting more distinct microphase separation, while further increase to 90% led to reduced d-spacing due to tighter chain packing and entanglement. These trends were consistent with AFM observations, confirming the influence of chain structure and phase domain organization on microphase-separated morphology.

#### 3.4.3. Density and Fractional Free Volume Analysis

The density and fractional free volume (FFV) of the QTB series membranes were measured to further evaluate the effects of alkyl chain length and alkylation degree on the membrane microstructure. The results were summarized in Table 1. For membranes with different alkyl chain lengths, both the membrane density and FFV initially increased with increasing chain length and then decreased at longer chains (C_4_). Similarly, for membranes with different alkylation degrees, the density and FFV increased with increasing alkylation degree and subsequently decreased at higher alkylation levels (70%). Notably, the QTB-C_4_-70% membrane exhibited a relatively low density of 0.99 g/cm^3^ and a high FFV of approximately 17%. These trends were consistent with the AFM and SAXS observations, confirming the close relationship among chain structure, microphase separation, and free volume distribution.

### 3.5. Gas Permeation Performance of TB and QTB Membranes

**Figure 9:**
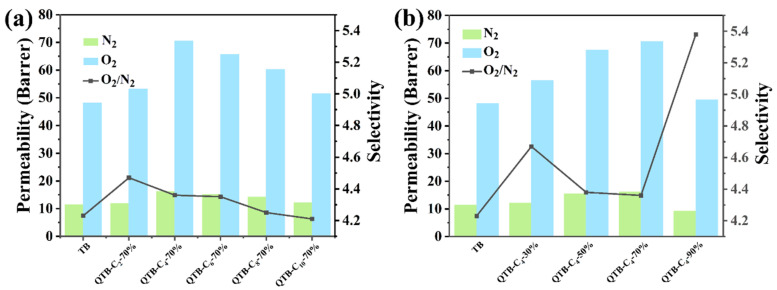
Gas permeability and selectivity of pristine TB and QTB membranes. (**a**) grafted with different lengths of iodoalkanes; (**b**) grafted with different ratios of iodobutane. 1 Barrer = 10^−10^ cm^3^ (STP) cm/(cm^2^ s cm Hg), the data were measured at 25 °C and 50 psi.

Before evaluating the performance of TB and QTB membranes, the membrane samples were initially immersed in methanol for 24 h, followed by vacuum drying at 100 °C to remove any residual solvent. Single-gas permeability measurements were conducted for N_2_ and O_2_, considering the different dynamic diameters of the gas molecules (N_2_ > O_2_). First, the effect of quaternization with iodoalkanes of varying carbon chain lengths, at a constant degree of quaternization, was investigated. The results are shown in Figure 9a. The O_2_ permeability increased from 48.2 to 70.6 Barrer as the carbon chain length increased from the pristine TB to QTB-C_4_-70%. However, further increasing the carbon chain length from QTB-C_4_-70% to QTB-C_10_-70% led to a decrease in O_2_ permeability to 51.5 Barrer. The QTB-C_4_-70% membrane, grafted with four-carbon chains, exhibited the highest O_2_ permeability, making it a suitable candidate for further study.

Next, the effect of different degrees of quaternization with varying amounts of iodobutane was investigated, with the results shown in Figure 9b. Compared with the pristine TB membrane (48.2 Barrer), the O_2_ permeability of QTB membranes increased from 60.7 to 70.6 Barrer as the degree of quaternization increased from 30% to 70%. This enhancement was attributed to two factors: the introduction of hydrophobic groups, which promoted O_2_ transport, and the formation of naturally ordered nanochannels by the hydrophilic tertiary amine groups and hydrophobic side chains, which facilitated gas diffusion. However, when the grafting ratio increased to 90%, the O_2_ permeability decreased to 49.5 Barrer, likely due to excessive side chains interlocking and disrupting molecular packing. Overall, the QTB-C_4_-70% membrane exhibited the highest O_2_ permeability, approximately 46% higher than that of the TB membrane. Meanwhile, the O_2_/N_2_ selectivity increased from 4.24 for the pristine TB membrane to 5.38 for QTB-C_4_-90%, corresponding to a 27% improvement. These trends in oxygen permeability were consistent with the AFM observations. In summary, the QTB membrane with a grafting ratio of 70% exhibited the highest O_2_ permeability coefficient (70.62 Barrer).

**Figure 10:**
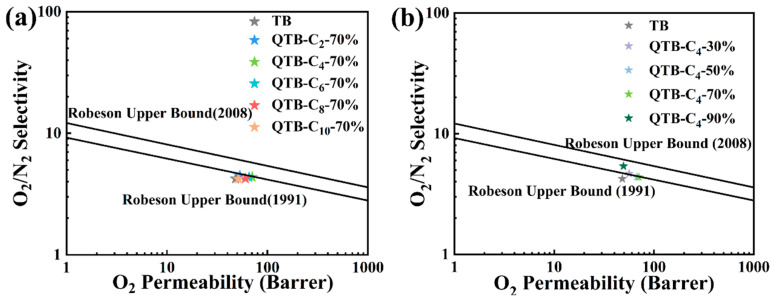
Robeson upper bound comparison of pristine TB and QTB membranes (**a**) grafted with different lengths of iodoalkanes; (**b**) grafted with different ratios of iodobutane for O_2_/N_2_ (at 50 psi, 25 °C).

The performance of gas separation membranes was often measured by the Robeson’s upper bound. The relationship between O_2_/N_2_ permeability (P) and selectivity (α), which were critical parameters for evaluating gas separation performance, was presented in Figure 10. Compared with TB membrane, the gas permeability and selectivity of the QTB membrane quaternized with different concentrations of iodoalkanes both increased. Simultaneously, nearly all data points reached the 1991 Robeson upper bound, while QTB-C_4_-90% was positioned in close proximity to the 2008 Robeson upper bound in Figure 10. These findings demonstrated that the gas separation performance of TB membrane was adjusted by grafting hydrophobic groups. As summarized in Table 2, the O_2_ separation performance of the QTB-C_4_-70% membrane was comparable to that of previously reported TB-based and polyimide membranes. While the performance was not exceptional, the proposed approach provided an effective strategy for constructing microphase-separated membranes.

### 3.6. Pressure Stability of QTB Membranes

**Figure 11:**
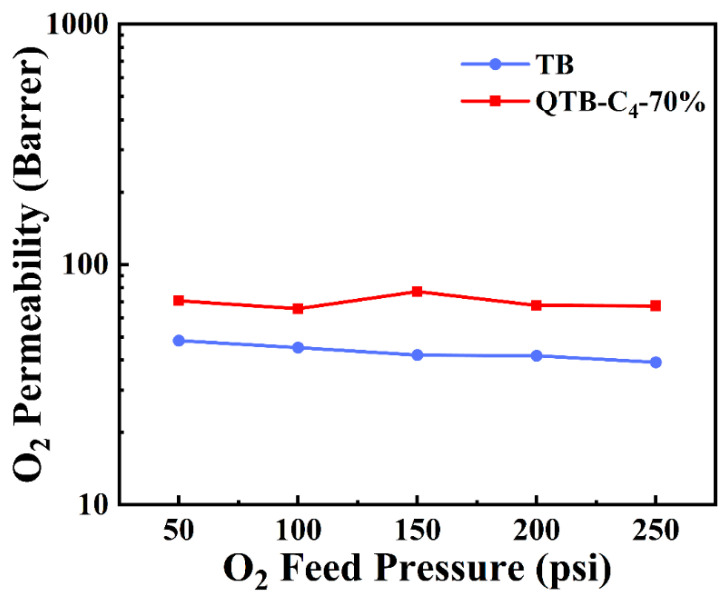
O_2_ permeability as a function of feed pressure for TB and QTB-C_4_-70% membranes (at 25 °C).

In membrane material research, pressure stability is an important indicator for evaluating the performance of gas separation membranes. Polymer membranes may exhibit pressure-dependent transport behavior under elevated feed pressures due to changes in chain packing or membrane compaction. To evaluate the pressure stability of QTB membranes under O_2_ exposure, the permeability of TB and QTB membranes was measured at varying O_2_ feed pressures using the constant-volume method. As shown in Figure 11, the O_2_ permeability of both TB and QTB membranes shows no pronounced increasing trend with increasing feed pressure, indicating stable gas transport behavior and good resistance to pressure-induced effects.

### 3.7. Effect of Temperature on the QTB Membranes

**Figure 12:**
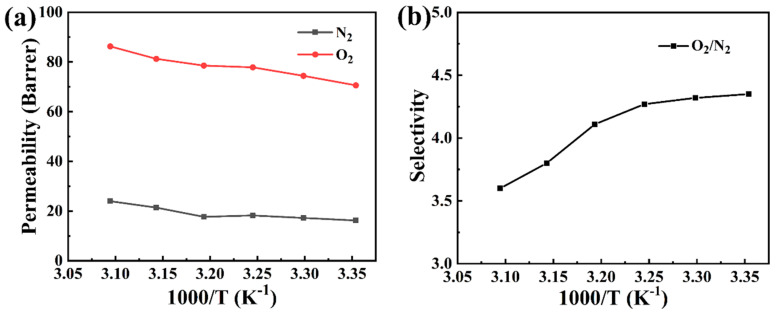
(**a**) Gas permeability of QTB-C_4_-70% membrane at different temperatures (at 50 psi); (**b**) selectivity of QTB-C_4_-70% membrane at different temperature (at 50 psi).

The performance of QTB-C_4_-70% membrane was investigated by varying the temperature (25 °C to 50 °C) at a constant feed pressure (50 psi). The gas permeability declined with decreasing temperature, while the selectivity experienced a rise in Figure 12a. This observed trend indicated that the performance of all gases exhibited temperature dependence following the Arrhenius-type relationship, aligning with the behavior seen in nonporous polymeric membranes, including polyimide and poly (ether block amide) gas separation membranes [60]. The proportional relationship between permeability and temperature could be explained by the following factors to explain it. (1) Accelerated gas molecule movement: Higher temperatures increased the kinetic energy of gas molecules, enhancing their diffusion rates. (2) Increased polymer chain mobility: Elevated temperatures enhance polymer chain flexibility and expand the chain spacing, facilitating gas molecule diffusion.

As illustrated in Figure 12b, within the temperature range of 25–50 °C, the permeability of N_2_ rose by about 48%, while the permeability of O_2_ increased by about 22%. This disparity led to a decline in selectivity with increasing temperature. The results showed that the QTB-C_4_-70% membrane exhibited excellent gas separation performance at low temperature, making it more suitable for use at lower temperature.

The reduction in selectivity at higher temperatures was primarily due to the unbalanced increase in gas permeation rates. The activation energy of permeation (*E_p_*) of typical membranes was calculated by the following Arrhenius equation:

$$J\;=\;J_{0}exp\left(-\frac{E_{p}}{RT}\right)$$

*J* represented gas flux, while *J*_0_ denoted the pre-exponential factor, which was temperature-independent and shared the same unit as the flux, *R* stood for the gas constant, and *T* corresponded to the absolute temperature. The equation provided remained applicable within a temperature range that avoided inducing significant thermal transitions in the polymer.

### 3.8. Effect of Days on the QTB Membranes

**Figure 13:**
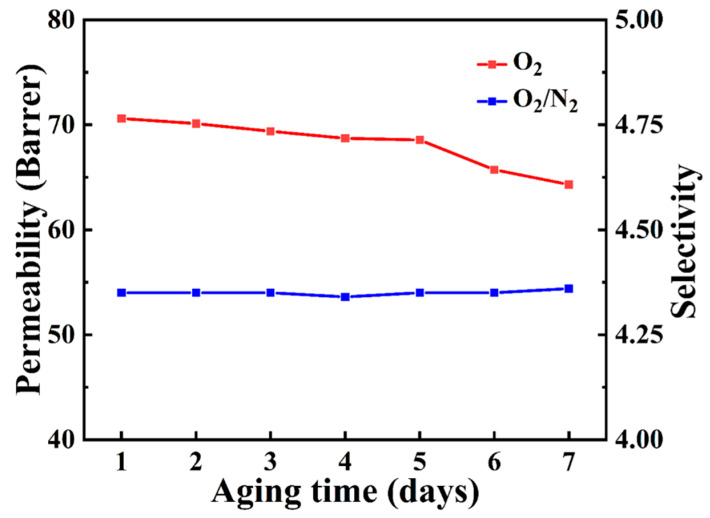
O_2_ permeability and selectivity of O_2_/N_2_ versus aging time of QTB-C_4_-70% membrane (at 25 °C, 50 psi).

The performance of the QTB-C_4_-70% membrane in separation was systematically evaluated under controlled conditions of 25 °C and 50 psi feed pressure. The oxygen permeation flux exhibited a gradual decline over the experimental duration, decreasing from an initial value of 70.60 Barrer to 64.32 Barrer after 7 days of continuous operation illustrated in Figure 13. This reduction in flux can be attributed to potential membrane compaction or minor physical aging effects under the applied pressure. Notably, the O_2_/N_2_ selectivity remained remarkably stable throughout the testing period, maintaining a consistent value of 4.35 ± 0.1. Overall, these results indicate that the QTB-C4-70% membrane exhibits good short-term aging stability, characterized by only a moderate decline in permeability while preserving stable gas separation selectivity under the investigated conditions.

## 4. Conclusions

In summary, a TB polymer was successfully synthesized via an aromatic nucleophilic substitution polycondensation process at low temperature. By employing a hydrophobic side-chain quaternization strategy, QTB gas separation membranes with well-defined microphase-separated structures were developed. Systematic variation in alkyl chain length and quaternization degree enabled control over the microphase separation morphology. The grafting of hydrophobic side chains onto the TB polymer induced a well-defined phase-separated microstructure in the QTB membranes, as confirmed by AFM and SAXS analyses. This structural modification resulted in a significant enhancement of gas permeability while maintaining moderate improvements in selectivity. Comparative gas permeation tests showed that all quaternized QTB membranes, regardless of iodoalkane type or concentration, outperformed the pristine TB membrane in both permeability and selectivity. Notably, the QTB-C_4_-70% membrane exhibited exceptional oxygen permeability, with most data points approaching or exceeding the 1991 Robeson upper bound. The material exhibited good pressure stability, as the O_2_ permeability showed no significant variation over the investigated pressure range. In temperature-dependent measurements, the O_2_ permeability decreased, while the selectivity was enhanced. By taking advantage of the reactivity of the tertiary amine groups in the TB polymer, a polarity difference between the main chain hydrophilic quaternary ammonium groups and the side chain hydrophobic alkyl groups was ingeniously constructed. This molecular design provided a structural basis for forming regular microphase separation channels. The performance of TB-based copolymers still has room for improvement, and the microphase separation control strategy established in this study provides theoretical guidance and technical approaches for developing a new generation of high-performance gas separation membrane materials. Through further optimization of polymer structure design and expansion of the TB-based polymer system, breakthrough progress in membrane material performance is expected.

## Figures and Tables

**Table 1:** Density and Fractional Free Volume of QTB Series Membranes.

Membrane	ρ_0_ (g/cm^3^)	ρ (g/cm^3^)	FFV
QTB-C_2_-70%	1.25	1.05	0.16
QTB-C_4_-70%	1.19	0.99	0.17
QTB-C_6_-70%	1.23	1.06	0.14
QTB-C_8_-70%	1.20	1.07	0.11
QTB-C_10_-70%	1.17	1.09	0.07
QTB-C_4_-30%	1.18	1.09	0.08
QTB-C_4_-50%	1.16	1.01	0.13
QTB-C_4_-70%	1.19	0.99	0.17
QTB-C_4_-90%	1.20	1.08	0.10

**Table 2:** Gas separation performance of TB-based or Polyimide membranes in the literature.

Membrane	Permeability		Selectivity	Refer.
	N_2_	O_2_	O_2_/N_2_	
QTB-C_4_-70%	16.2	70.6	4.4	This work
TB (10.0 wt%)	2.8	27.9	10.0	[53]
PI/TB	4.4	21.3	4.8	[54]
PIM-PI-TB-2	34.0	123.0	3.6	[55]
PI@Trip-TB (1:10)	5.6	36.2	6.5	[56]
6FTB3-TR450	15.0	52.0	3.5	[57]
PI-TB-5	1.9	4.9	2.6	[58]
CoPI-TB-6	16.0	73.0	4.6	[58]
Matrimid^®^ 5218	2.1	13.0	6.2	[59]
DOCDA	1.3	8.7	6.7	[59]

## Data Availability

The original contributions presented in this study are included in the article. Further inquiries can be directed to the corresponding authors.

## References

[1] Wang W., Li F., Wang H. (2023). Numerical simulation study on the effect of different oxygen-enrichment atmospheres on diesel combustion. Energy.

[2] Anwar M.N., Fayyaz A., Sohail N.F., Khokhar M.F., Baqar M., Khan W.D., Rasool K., Rehan M., Nizami A.S. (2018). CO_2_ capture and storage: A way forward for sustainable environment. J. Environ. Manag..

[3] Arnaiz del Pozo C., Sánchez-Orgaz S., Navarro-Calvo A., Jiménez Álvaro Á., Cloete S. (2024). Integration of Chemical Looping Combustion in the Graz Power Cycle. Energies.

[4] Hazarika G., Ingole P.G. (2024). Polymer-based hollow fiber membranes: A modern trend in gas separation technologies. Mater. Today Chem..

[5] Niu Z., He N., Yao Y., Ma A., Zhang E., Cheng L., Li Y., Lu X. (2024). Mixed matrix membranes for gas separations: A review. Chem. Eng. J..

[6] Alqaheem Y., Alomair A.A. (2020). Sealing perovskite membranes for long-term oxygen separation from air. Chem. Pap..

[7] Han Y., Ho W.S.W. (2022). Moving beyond 90% Carbon Capture by Highly Selective Membrane Processes. Membranes.

[8] Han Y., Ho W.S.W. (2018). Recent advances in polymeric membranes for CO_2_ capture. Chin. J. Chem. Eng..

[9] Liu Y., Zhao G., Tong F., Li Z., Wang L., Fang C., Lei L., Xu Z. (2025). Sandwich-like multilayer hollow fiber carbon membranes for gas separations. J. Membr. Sci..

[10] Zhang Y., Yin B.H., Huang L., Ding L., Lei S., Telfer S.G., Caro J., Wang H. (2025). MOF membranes for gas separations. Prog. Mater. Sci..

[11] Ge C., Sheng M., Yuan Y., Shi F., Yang Y., Zhao S., Wang J., Wang Z. (2023). Recent advances of the interfacial polymerization process in gas separation membranes fabrication. J. Membr. Sci..

[12] Yang H.-C., Hou J., Chen V., Xu Z.-K. (2016). Surface and interface engineering for organic–inorganic composite membranes. J. Mater. Chem. A.

[13] Seoane B., Coronas J., Gascon I., Benavides M.E., Karvan O., Caro J., Kapteijn F., Gascon J. (2015). Metal–organic framework based mixed matrix membranes: A solution for highly efficient CO_2_ capture?. Chem. Soc. Rev..

[14] Dai Z., Noble R.D., Gin D.L., Zhang X., Deng L. (2016). Combination of ionic liquids with membrane technology: A new approach for CO_2_ separation. J. Membr. Sci..

[15] Cheng Y., Ying Y., Japip S., Jiang S.-D., Chung T.-S., Zhang S., Zhao D. (2018). Advanced Porous Materials in Mixed Matrix Membranes. Adv. Mater..

[16] Zhu J., Yuan S., Wang J., Zhang Y., Tian M., Van der Bruggen B. (2020). Microporous organic polymer-based membranes for ultrafast molecular separations. Prog. Polym. Sci..

[17] Melone L., Brunetti A., Giorno L., Barone M., Barbieri G. (2020). Trichloroethylene/Nitrogen Mixture Separation via membrane operations: Comparison with traditional technologies. Sep. Purif. Technol..

[18] Liu J., Fulong C.R.P., Hu L., Huang L., Zhang G., Cook T.R., Lin H. (2020). Interpenetrating networks of mixed matrix materials comprising metal-organic polyhedra for membrane CO_2_ capture. J. Membr. Sci..

[19] Wang Y., Ghanem B.S., Han Y., Pinnau I. (2022). State-of-the-art polymers of intrinsic microporosity for high-performance gas separation membranes. Curr. Opin. Chem. Eng..

[20] Zhao S., Liao J., Li D., Wang X., Li N. (2018). Blending of compatible polymer of intrinsic microporosity (PIM-1) with Tröger’s Base polymer for gas separation membranes. J. Membr. Sci..

[21] Feng X., Zhu J., Jin J., Wang Y., Zhang Y., Van der Bruggen B. (2024). Polymers of intrinsic microporosity for membrane-based precise separations. Prog. Mater. Sci..

[22] Wang A., Zhao M., Weng Y., Zhang C. (2025). Nonlinear learning-enhanced MLP-driven design of PIMs gas separation membranes beyond Robeson upper bounds. J. Membr. Sci..

[23] Xiao Y., Zhang L., Xu L., Chung T.-S. (2017). Molecular design of Tröger’s base-based polymers with intrinsic microporosity for gas separation. J. Membr. Sci..

[24] Carta M., Malpass-Evans R., Croad M., Rogan Y., Lee M., Rose I., McKeown N.B. (2014). The synthesis of microporous polymers using Tröger’s base formation. Polym. Chem..

[25] Wang X., Wu L., Li N., Fan Y. (2021). Sealing Tröger base/ZIF-8 mixed matrix membranes defects for improved gas separation performance. J. Membr. Sci..

[26] Lei X., Zhang Z., Xiao Y., Yu Q., Liu Y., Ma X., Zhang Q. (2025). Tröger’s Base Polyimide Membranes with Enhanced Mechanical Robustness for Gas Separation. Polymers.

[27] Sun Y., Bi X., Bai L., Li T., Fan F., Gao Z., Liu Y., Guan J., Sun F., Li H. (2025). Mixed matrix membranes incorporating bromine functionalized UiO-66 in the Tröger’s base polymer for enhanced CO_2_ capture. Chem. Eng. Sci..

[28] Hu X., Lee W.H., Bae J.Y., Zhao J., Kim J.S., Wang Z., Yan J., Lee Y.M. (2020). Highly permeable polyimides incorporating Tröger’s base (TB) units for gas separation membranes. J. Membr. Sci..

[29] Hu X., Lee W.H., Zhao J., Bae J.Y., Kim J.S., Wang Z., Yan J., Zhuang Y., Lee Y.M. (2020). Tröger’s Base (TB)-containing polyimide membranes derived from bio-based dianhydrides for gas separations. J. Membr. Sci..

[30] Chen Z., Meng X., Fan Y., Li N., Wu L., Luo S., Xie W., Wang C. (2023). Two morphology distinct fillers with chemical similarity incorporated into Tröger’ base polymers towards enhanced gas separation. J. Membr. Sci..

[31] Yue J., Hou J., Li Y., Yang Y., Han L., Sun S., Li J. (2022). Branched Tröger’s base polymer membranes for gas separation. Polymer.

[32] Yang Z., Guo R., Malpass-Evans R., Carta M., McKeown N.B., Guiver M.D., Wu L., Xu T. (2016). Highly Conductive Anion-Exchange Membranes from Microporous Tröger’s Base Polymers. Angew. Chem. Int. Ed..

[33] Swaidan R., Ghanem B., Pinnau I. (2015). Fine-Tuned Intrinsically Ultramicroporous Polymers Redefine the Permeability/Selectivity Upper Bounds of Membrane-Based Air and Hydrogen Separations. ACS Macro Lett..

[34] Wang Z., Wang D., Jin J. (2014). Microporous Polyimides with Rationally Designed Chain Structure Achieving High Performance for Gas Separation. Macromolecules.

[35] Swaidan R., Al-Saeedi M., Ghanem B., Litwiller E., Pinnau I. (2014). Rational Design of Intrinsically Ultramicroporous Polyimides Containing Bridgehead-Substituted Triptycene for Highly Selective and Permeable Gas Separation Membranes. Macromolecules.

[36] Rogan Y., Malpass-Evans R., Carta M., Lee M., Jansen J.C., Bernardo P., Clarizia G., Tocci E., Friess K., Lanč M. (2014). A highly permeable polyimide with enhanced selectivity for membrane gas separations. J. Mater. Chem. A.

[37] Ghanem B.S., Swaidan R., Ma X., Litwiller E., Pinnau I. (2014). Energy-Efficient Hydrogen Separation by AB-Type Ladder-Polymer Molecular Sieves. Adv. Mater..

[38] Carta M., Croad M., Malpass-Evans R., Jansen J.C., Bernardo P., Clarizia G., Friess K., Lanč M., McKeown N.B. (2014). Triptycene Induced Enhancement of Membrane Gas Selectivity for Microporous Tröger’s Base Polymers. Adv. Mater..

[39] Carta M., Croad M., Jansen J.C., Bernardo P., Clarizia G., McKeown N.B. (2014). Synthesis of cardo-polymers using Tröger’s base formation. Polym. Chem..

[40] Wu W., Niu H., Lai S., Liu C., Zhou L., Huang X. (2022). Synthesis, characterization, and gas separation properties of novel fluorinated co-polyimides with bulky side groups. Polymer.

[41] Comesaña-Gándara B., Chen J., Bezzu C.G., Carta M., Rose I., Ferrari M.-C., Esposito E., Fuoco A., Jansen J.C., McKeown N.B. (2019). Redefining the Robeson upper bounds for CO_2_/CH_4_ and CO_2_/N_2_ separations using a series of ultrapermeable benzotriptycene-based polymers of intrinsic microporosity. Energy Environ. Sci..

[42] Bernardo P., Bazzarelli F., Tasselli F., Clarizia G., Mason C.R., Maynard-Atem L., Budd P.M., Lanč M., Pilnáček K., Vopička O. (2017). Effect of physical aging on the gas transport and sorption in PIM-1 membranes. Polymer.

[43] Zheng J., Wang C., Jing C., Wu Y., Zhang K., Luo S. (2025). Pentiptycene-based ladder polymers for membrane gas separation. React. Funct. Polym..

[44] Wang Z.G., Liu X., Wang D., Jin J. (2014). Tröger’s base-based copolymers with intrinsic microporosity for CO_2_ separation and effect of Tröger’s base on separation performance. Polym. Chem..

[45] Yuan L., Zhu P., Wang Y., Li X., Yang Y., Du H., Dong X., Wang D. (2024). Gas transport properties of poly(ether-b-amide) segmented copolymers: The role of the degree of microphase separation in amorphous regions. J. Membr. Sci..

[46] Kim K.J., Chae Y., An S.J., Jo J.H., Park S., Chi W.S. (2023). Microphase-separated morphology controlled polyimide graft copolymer membranes for CO_2_ separation. Sep. Purif. Technol..

[47] Sun L., Xu W., Zhang H., Chu J., Wang M., Song K., Wu W., Li J., Wang Y., Pinnau I. (2025). In-Situ Formation of Three-Dimensional Network Intrinsic Microporous Ladder Polymer Membranes with Ultra-High Gas Separation Performance and Anti-Trade-Off Effect. Angew. Chem. Int. Ed..

[48] Wu C., Wang Z., Liu S., Xie Z., Chen H., Lu X. (2018). Simultaneous permeability, selectivity and antibacterial property improvement of PVC ultrafiltration membranes via in-situ quaternization. J. Membr. Sci..

[49] Xu Z., Liao J., Tang H., Efome J.E., Li N. (2018). Preparation and antifouling property improvement of Tröger’s base polymer ultrafiltration membrane. J. Membr. Sci..

[50] Wang A., Liao J., Wu X., Meng X., Ma J., Peng S., Shen Y., Gao C. (2024). PI@TB blend membranes containing amorphous carbon for gas separation. Sep. Purif. Technol..

[51] Chen J., Shen C., Gao S. (2024). Polyepichlorohydrin grafted poly(phenylene oxide) to construct hydrophilic and hydrophobic microphase separation anion exchange membrane. React. Funct. Polym..

[52] Park C.H., Lee J.H., Jung J.P., Lee W., Ryu D.Y., Kim J.H. (2018). Orientation of an Amphiphilic Copolymer to a Lamellar Structure on a Hydrophobic Surface and Implications for CO_2_ Capture Membranes. Angew. Chem. Int. Ed..

[53] Sun Y., Gao Z., Bai L., Li T., Fan F., Sun F., Liu Y., Guan J., He G., Ma C. (2025). Hydrogen-bond crosslinking of Tröger’s base polymer membranes for enhanced gas selectivity and plasticization resistance. J. Membr. Sci..

[54] Zhou J., Zhu X., Hu J., Liu H., Hu Y., Jiang J. (2014). Mechanistic insight into highly efficient gas permeation and separation in a shape-persistent ladder polymer membrane. Phys. Chem. Chem. Phys..

[55] Ghanem B., Alaslai N., Miao X., Pinnau I. (2016). Novel 6FDA-based polyimides derived from sterically hindered Tröger’s base diamines: Synthesis and gas permeation properties. Polymer.

[56] Chen X., Zhang Z., Wu L., Fan Y., Tang H., Li N. (2022). Hydrogen bonding-induced 6FDA-DABA/TB polymer blends for high performance gas separation membranes. J. Membr. Sci..

[57] Song Y.-x., Tang A., Zhang J.-l., Dong J., Zhao X., Zhang Q.-h. (2025). Preparation of Thermally Rearranged Poly(benzoxazole-co-imide) Membranes Containing Tröger’s Base Structure and Their Gas Separation Performances. Acta Polym. Sin..

[58] Hu X., Miao J., Pang Y., Zhao J., Lu Y., Guo H., Wang Z., Yan J. (2022). Synthesis, microstructures, and gas separation performance of norbornyl bis-benzocyclobutene-Troger’s base polymers derived from pure regioisomers. Polym. Chem..

[59] Tsegay Tikue E., Kyung Kang S., Ha Kim M., Chul Kwak W., An I., Yang S., Kim J.-H., Soo Lee P. (2024). Mixed matrix membrane based on DOCDA polyimide with twisted kink structure and zeolite 4A for energy-efficient oxygen purification. J. Ind. Eng. Chem..

[60] Liu L., Qiu W., Sanders E.S., Ma C., Koros W.J. (2016). Post-combustion carbon dioxide capture via 6FDA/BPDA-DAM hollow fiber membranes at sub-ambient temperatures. J. Membr. Sci..

